# Gut microbiota and metabolites of α-synuclein transgenic monkey models with early stage of Parkinson’s disease

**DOI:** 10.1038/s41522-021-00242-3

**Published:** 2021-09-02

**Authors:** Yaping Yan, Shuchao Ren, Yanchao Duan, Chenyu Lu, Yuyu Niu, Zhengbo Wang, Briauna Inglis, Weizhi Ji, Yun Zheng, Wei Si

**Affiliations:** State Key Laboratory of Primate Biomedical Research, Institute of Primate Translational Medicine, Kunming University of Science and Technology, Kunming, China

**Keywords:** Microbiota, Metagenomics

## Abstract

Parkinson’s disease (PD) is the second most prevalent neurodegenerative disease. However, it is unclear whether microbiota and metabolites have demonstrated changes at early PD due to the difficulties in diagnosis and identification of early PD in clinical practice. In a previous study, we generated A53T transgenic monkeys with early Parkinson’s symptoms, including anxiety and cognitive impairment. Here we analyzed the gut microbiota by metagenomic sequencing and metabolites by targeted gas chromatography. The gut microbiota analysis showed that the A53T monkeys have higher degree of diversity in gut microbiota with significantly elevated Sybergistetes, Akkermansia, and *Eggerthella lenta* compared with control monkeys. Prevotella significantly decreased in A53T transgenic monkeys. Glyceric acid, L-Aspartic acid, and p-Hydroxyphenylacetic acid were significantly elevated, whereas Myristic acid and 3-Methylindole were significantly decreased in A53T monkeys. Glyceraldehyde-3-phosphate dehydrogenase (GAPDH) (KO0131) and the oxidative phosphorylation reaction (KO2147) were significantly increased in metabolic pathways of A53T monkeys. Our study suggested that the transgenic A53T and α-syn aggregation may affect the intestine microbiota and metabolites of rhesus monkeys, and the identified five compositional different metabolites that are mainly associated with mitochondrial dysfunction may be related to the pathogenesis of PD.

## Introduction

Parkinson’s disease (PD) is the second most prevalent neurodegenerative disease^1,2^. The main characteristics of PD include the loss of dopaminergic neurons in the substantia nigra. As a neuronal protein, α-synuclein (α-syn) plays an important role in the formation of the Lewy body. The aggregation of α-syn particularly impacts dopaminergic neurons of substantia nigra pars compacta (SNpc) and is closely related to Parkinsonism. PD is typically characterized by a combination of both motor features (e.g., bradykinesia and tremor) and a range of nonmotor features (e.g., cognitive impairment, anxiety, constipation, and disturbed sleep); these nonmotor features can precede the manifestation of the motor syndrome. The earliest stages of PD can be difficult to recognize, as reflected by the long delay (average 10 years) that typically separates the person’s first noticeable symptom from the timing of diagnosis. Early symptoms include constipation, cognitive impairment, anxiety and disturbed sleep, asymmetric vague shoulder pain, or depression^3–6^. The most recent hypothesis is that PD may originate in the gut and spread to the brain through α-syn transmission, systemic inflammation, and increased permeability of the blood-brain barrier^7,8^. Translocation bacteria and inflammatory bacteria increase intestinal inflammatory response and oxidative stress, triggering the accumulation of α-syn in the enteric nervous system^9,10^. The latest research also demonstrated that the injection of α-syn into the intestinal wall of rodents caused propagation of α-syn from the gut to the brain and leads to Parkinsonism, the aggregated α-syn can reach the brain via the vagus nerve^11^. As a result, gut microbiota may be thought to be a factor in the occurrence of PD.

The gut microbiota regulate the host’s metabolites, which affect the host’s biochemical and physiological processes, thereby increase the host’s susceptibility to disease^12^. The host and its intestinal microbiota together produce a large number of small molecules that play a decisive role in the transmission of information between the host cell and the microbiota in the metabolism of food and xenobiotics. At present, it has been shown that short-chain fatty acids were significantly reduced in the blood of PD patients^13–15^. A computational modeling of the gut microbiota showed that the interconversion of methionine and cysteine via cystathionine was found to be different between the PD patients and non-PD patients^16^. However, currently no other metabolites have been identified and reported at early PD. Evidence from recent years also indicates that mitochondrial dysfunction is central to the pathogenesis of both the sporadic and familial PD^17,18^. New data suggests that α-syn can interact with mitochondria by binding to the mitochondrial outer membrane^19,20^. This also indicates a certain relationship between the α-syn and the mitochondria. Alpha-syn can disrupt the introduction of mitochondrial proteins and localize to mitochondrial outer membranes in PD and postmortem PD patients. The accumulation of α-syn in the outer membrane may interfere with the introduction mechanism of the protein but may also interfere with the homeostatic pathway of other mitochondria^19,21^.

Parkinson’s disease (PD) is an age-dependent neurodegenerative disease. The complexity of PD is accompanied by clinical challenges, including an inability to make a definitive diagnosis at the earliest stages of the disease and difficulties in the management of symptoms at later stages. Animal models that can accurately express the metabolic and histological features of human PD play import roles in the mechanism study and the development of drugs and therapeutic strategies. The rodent models of PD are hardly to mimic the pathologic characteristics and symptoms of PD patients ideally. The close evolutionary history makes the brains of monkey are far more similar to human than those of the mice^22^. Therefore, nonhuman primates especially the gene-modified models serve as the most ideal animal model for the research of human neurodegenerative diseases. Recently, more and more studies have confirmed the role of the brain-gut axis in the development of nervous system disease, including PD^9–11^.

The close evolutionary history makes the brains of monkey are far more similar to human than those of the mice. Therefore, nonhuman primates especially the gene-modified models serve as the most ideal animal model for the research of human neurodegenerative diseases. In our previous study, we have confirmed the positive expression of A53T in the substantia nigra of aborted A53T fetuses, and the A53T transgenic monkeys showed anxiety and cognitive impairment that presenting the symptoms of early PD^23^. As to our knowledge, there is no report studying the interaction between the PD and intestinal microbiota in nonhuman primates so far. Furthermore, whether the overexpress exogenous gene such A53T can drive the change of intestinal microbiota of monkeys is still unknown. These specific monkey models provide a unique opportunity for us to analyze the gut microbiota and metabolites in transgenic rhesus monkeys at early PD and disclose the relationship between the gut microbiota, metabolites, and PD. Since the occurrence of motor deficits in PD patients initializes at the middle and/or late stages of PD, we aim to reveal potential diagnostic markers for early-stage PD disease before the onset of dyskinesia via the analysis of the gut microbes and metabolites of the α-syn mutant monkeys.

## Results

### Phylogenetic profiles of gut microbes in A53T transgenic monkeys

**Fig. 1:**
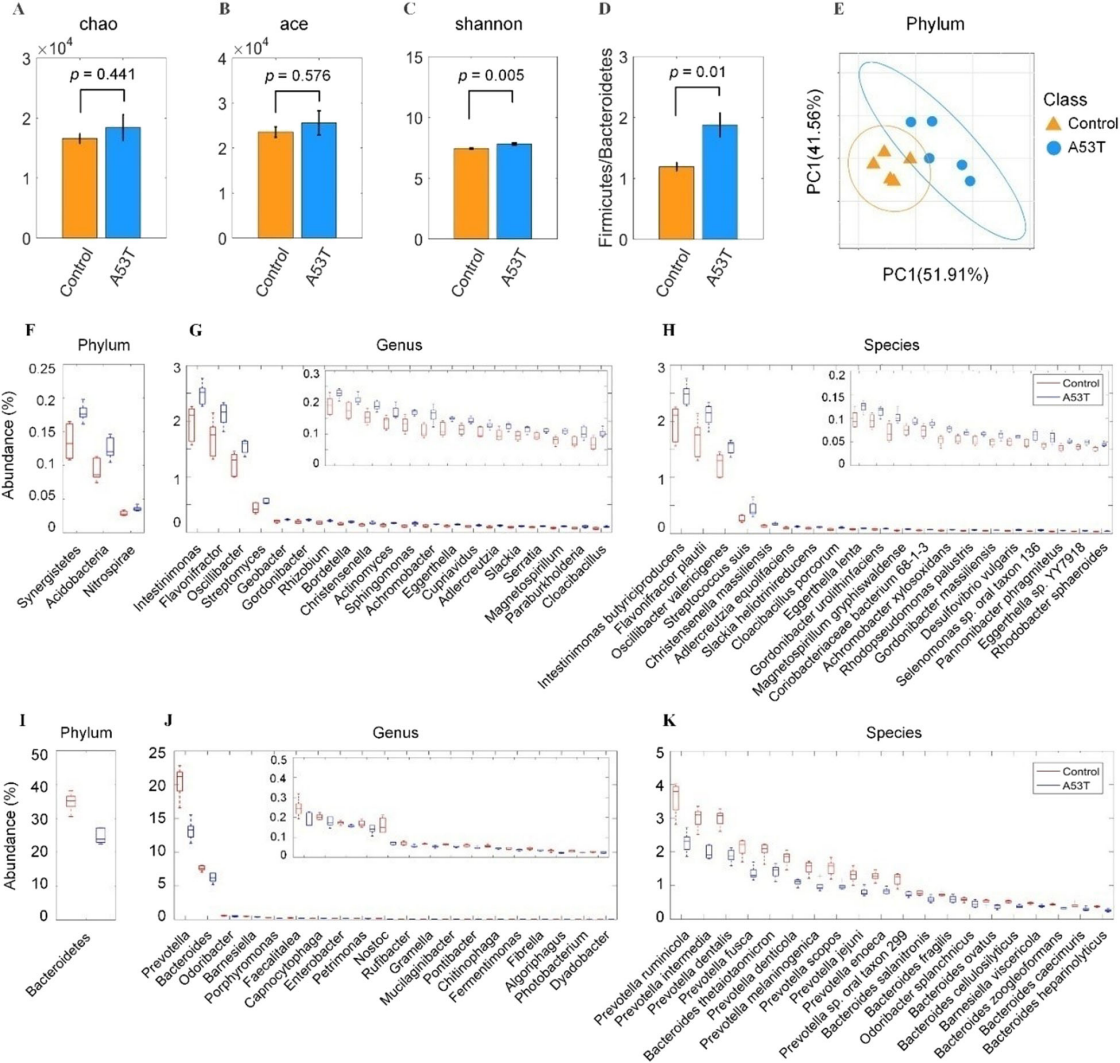
Phylogenetic profiles of gut microbes in A53T transgenic monkeys. **A–C** α diversity including Chao index, Ace index, Shannon index. Shannon index significantly increased in the A53T transgenic group compared to the control group (*p* < 0.005). **D** The ratio of Firmicutes to Bacteroidetes increased in the A53T transgenic group. **E** An obvious separation between the A53T transgenic group and the control group was observed by β diversity analysis. **F**, **G**, **H** The phylotypes significantly increased (*p* < 0.05) in the A53T transgenic monkeys at the phylum, genus, species levels. **I**, **J**, **K** The phylotypes significantly decreased in the A53T transgenic monkeys at the phylum, genus, and species levels.

Gut microbiota was characterized by metagenomics sequencing. Alpha diversity analysis including the Chao, Ace, and Shannon index showed that the fecal microbiota of A53T transgenic monkeys was more diverse compared to the controls, and the Shannon index have significant difference (*p* = 0.005) (Fig. 1A–C). The value of Firmicutes versus Bacteroidetes (F/B value) was increased in the A53T transgenic monkeys (Fig. 1D). Beta diversity by examining the unweighted Unifrac distance expounded that microbiota composition has an obvious separation between both groups (Fig. 1E).

Phylotypes with a median relative abundance larger than 0.01% of the total abundance in either the control monkeys or A53T monkeys were included for comparison. At the phylum level, Bacteroidetes and Firmicutes dominated the microbial communities of both groups. Compared with the control group, A53T monkeys had higher levels of Synergistetes, Acidobacteria, and Nitrospirae, but lower level of Bacteroidetes. At the genus level, 20 genera were observed to have significantly increased or decreased in the A53T monkeys compared with the control group. Prevotella and Bacteroides were the dominant phylotype in both groups, but significantly decreased in the A53T group. Of the remaining enriched genera, Intestinimonas, Flavonifractor, Oscillibacter, and Streptomyces were observed to have significantly increased in the A53T transgenic monkeys. In contrast, Odoribacter and Barnesiella significantly decreased in the A53T transgenic monkeys. At the species level, four species belong to Firmicutes, six species from Actinobacteria and four species belong to Proteobacteria were significantly increased in A53T monkeys (Supplementary Table 1). Of the top 20 decreased species in the A53T monkeys, ten species from Prevotella spp. and nine species from Bacteroides spp., which suggests that the two genera (Prevotella and Bacteroides) might play an important role in the onset of PD in monkeys (Fig. 1F–K).

### Microbiota from A53T transgenic and control monkeys produce discrete metabolite profiles and correlation analysis

**Fig. 2:**
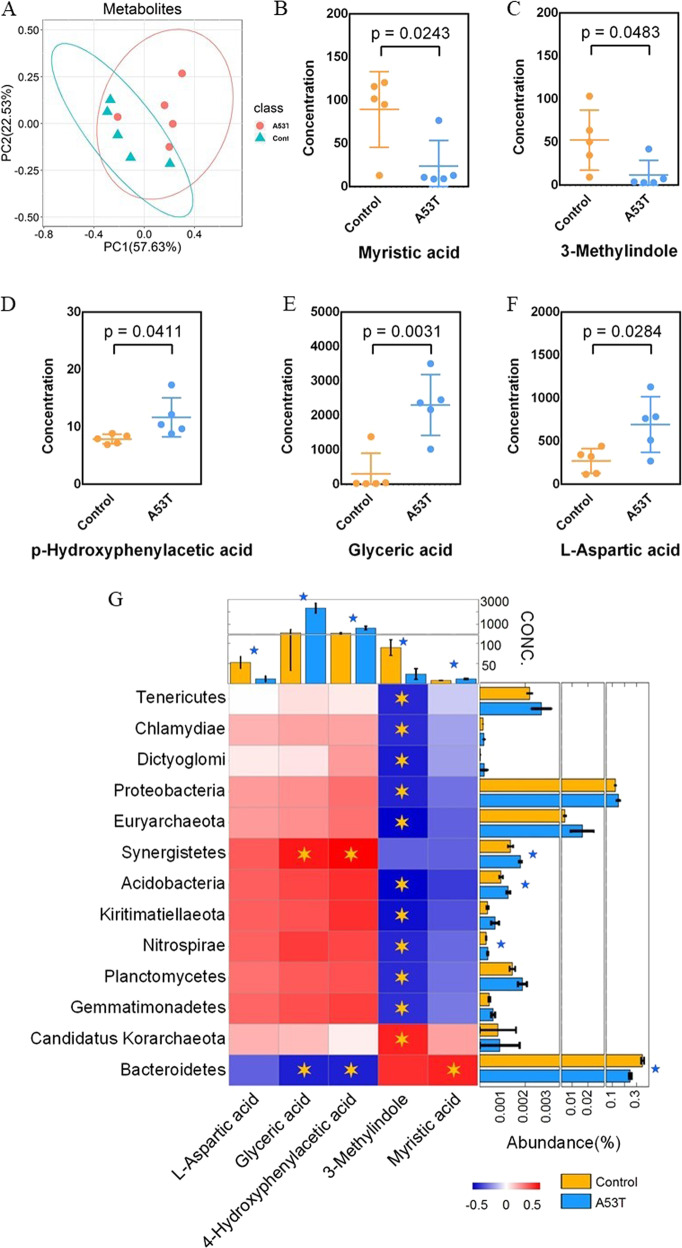
Microbiota from A53T transgenic and control monkeys produce discrete metabolite profiles and correlation analysis. **A** Metabolites had obviously divided between A53T transgenic monkeys and control monkeys. **B–C**) Myristic acid, 3-Methylindole significantly (*p* < 0.05) decreased in A53T group. **D–F** p-Hydroxyphenylacetic acid, Glyceric acid, and L-Aspartic acid significantly (*p* < 0.05) increased in A53T transgenic group. The concentrations of different metabolites in the A53T transgenic and control groups were compared with Welch’s *t* tests and the obtained p-values were corrected with the Benjamini Hochberg method. **G** Analyze the correlation between bacteria and metabolites. Glyceric acid and p-Hydroxyphenylacetic are positively correlated with Sybergistetes. Myristic acid and Bacteroidetes are significant positive correlated. *Candidatus Korarchaeota* and 3-Methylindole are positive correlated.

We performed targeted gas chromatography analyses of colon contents from A53T transgenic and control monkeys, and a total of 93 metabolites were found, mainly including amino acids and fatty acids (Supplementary Table 2). The abundance patterns of metabolites have significant differences between the two groups by PCoA analyze (Fig. 2A). Five different metabolites were found between the A53T transgenic and control monkeys that include 3-Methylindole, Glyceric acid, L-Aspartic, Myristic acid, and p-Hydroxyphenylacetic acid. Among the five different metabolites, 3-Methylindole and Myristic acid significantly decreased (multiple test corrected *p* < 0.05) (Fig. 2B, C) and Glyceric acid, L-Aspartic, and p-Hydroxyphenylacetic acid significantly increased (multiple test corrected *p* < 0.05) in A53T group compared to the control group (Fig. 2D–F).

Correlation analysis found that Glyceric acid and p-Hydroxyphenylacetic acid are positively correlated with Sybergistetes, which is significantly increased in A53T transgenic monkeys (multiple test corrected *p* < 0.05). Myristic acid and Bacteroidetes are significantly positively correlated. However, p-Hydroxyphenylacetic acid is significantly negatively correlated with Bacteroidetes, and Bacteroidetes is significantly decreased in A53T transgenic monkeys (multiple test corrected *p* < 0.05). *Candidatus Korarchaeota* and 3-Methylindole are significantly positively correlated (Fig. 2G).

### Integrated analysis of intestinal microbiota and metabolites

**Fig. 3:**
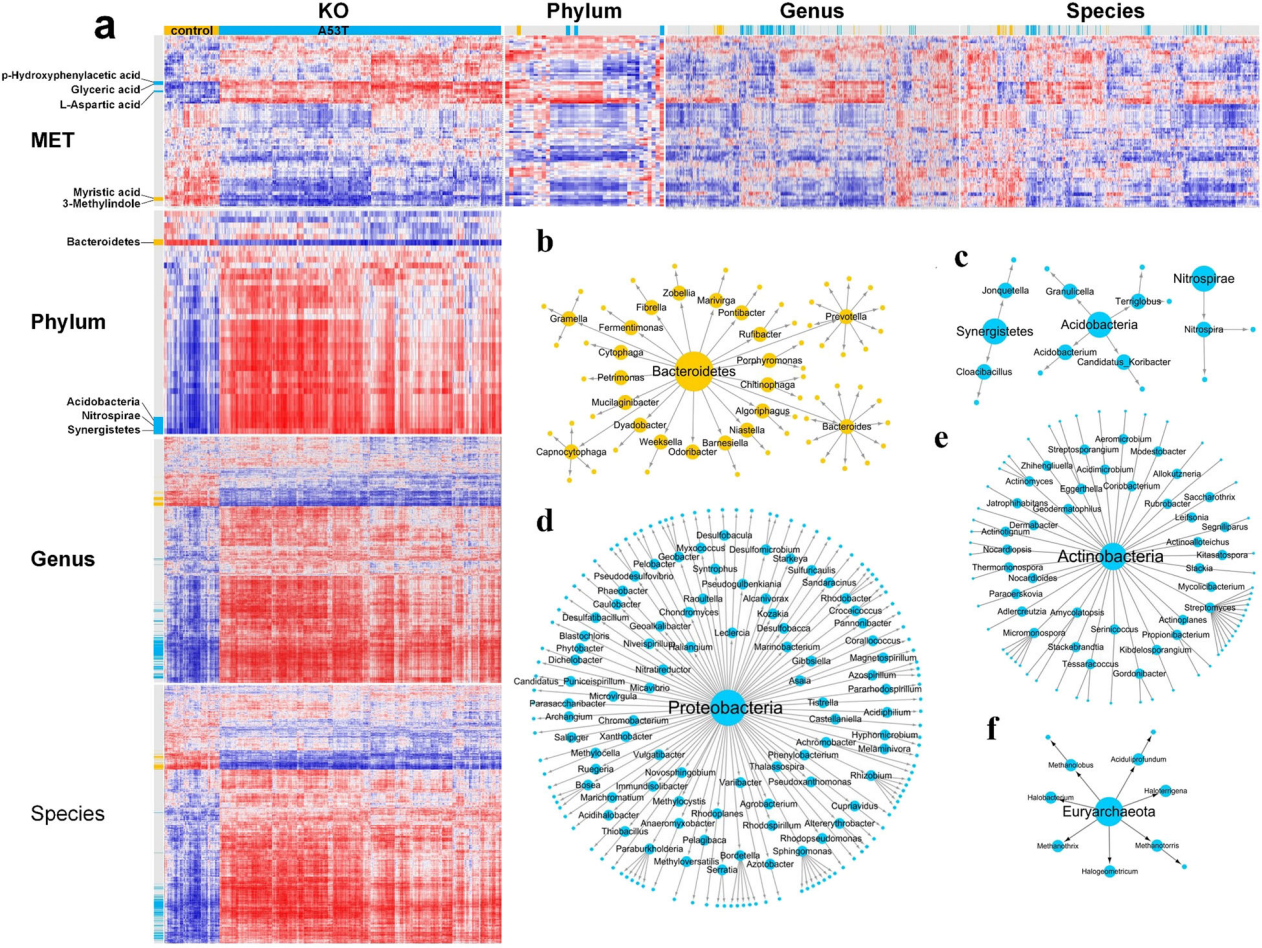
Integrated analysis of intestinal microbiota and metabolites. **a** The yellow/blue labels on the left side of the figure indicate the microbe showing significantly different enrichment in the control/A53T group (*p* < 0.05). **b**–**g** This difference has a distinct transmission relationship in the classification of part microbe (The direction of the phylum-genus-species is indicated by blue/yellow arrows). Yellow present increased in the A53T group. Blue present increased in the control group.

Since the metagenomics and metabolite profiles were produced from the same batch of samples, therefore we explored the potential relationship between the gut microbiota and metabolites. We performed a correlation analysis for the microbiota, metabolites, and functions. Significant differences in the microbiota, metabolites, and functions between the two groups were observed, and the changes showed similar trend with a good consistency (Fig. 3A). We made a network of six phyla, which are the most abundant in the two groups with consistency. Acidobacteria, Sybergistetes, and Nitrospirae significantly increased in A53T transgenic monkeys. In contrast, Bacteroidetes significantly decreased in A53T transgenic monkeys. Proteobacteria and Actinobacteria were not significantly different between the two groups but 80 genera from Proteobacteria (about 80%) and 36 genera from Actinobacteria (about 70%) were significantly elevated in A53T transgenic monkeys (Fig. 3B–G).

**Fig. 4:**
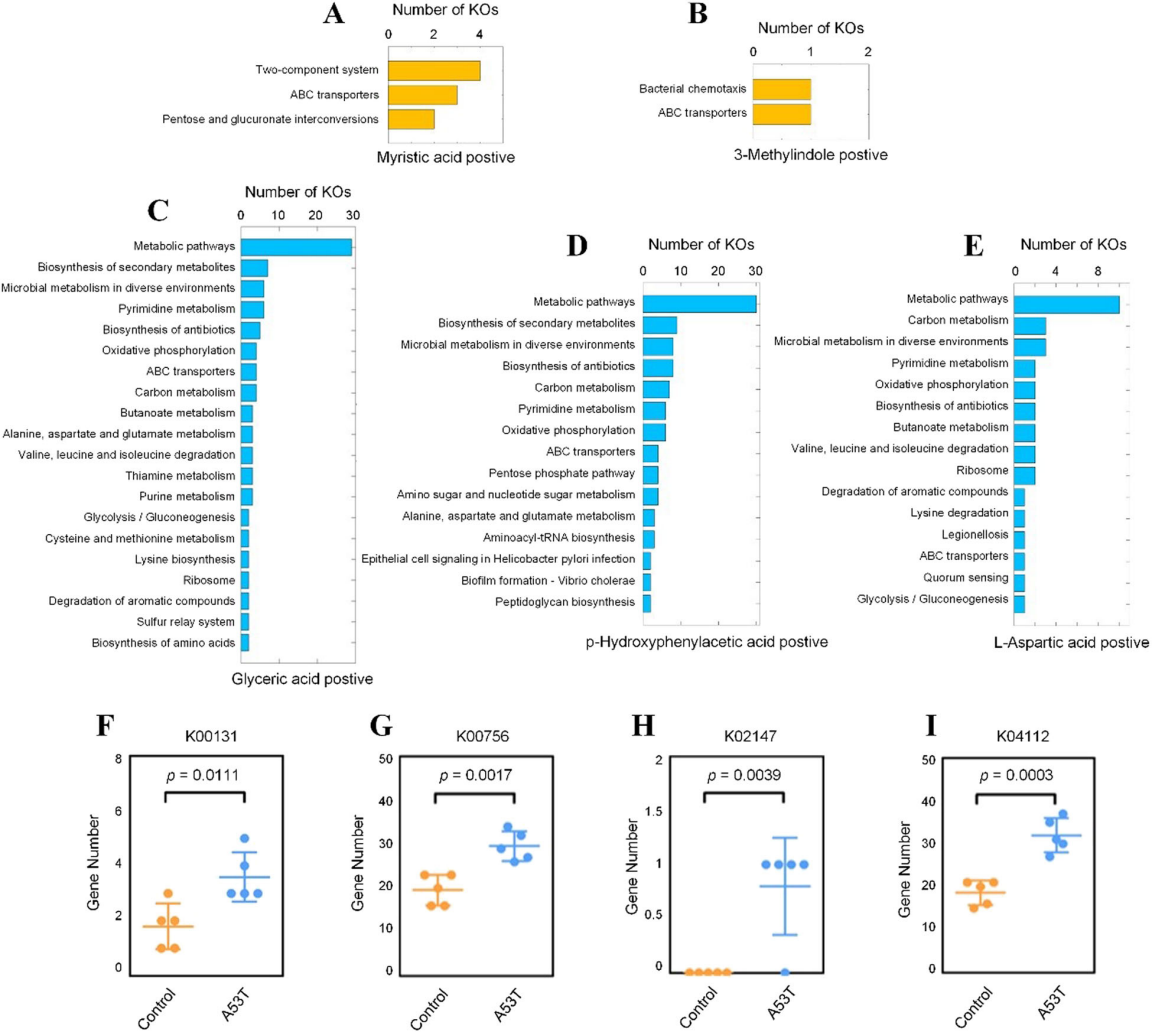
Analysis of enriched pathways for the five differential metabolites. **A–B** Metabolite-enriched pathways significantly (*p* < 0.05) associated with control monkeys. **C–E** Metabolite-enriched pathways significantly (*p* < 0.05) associated with A53T transgenic monkeys. **F–I** Four KOs from metabolic pathways that are significantly (*p* < 0.05) elevated in the transgenic A53T monkeys.

In order to find the associated pathways, we performed a functional analysis of the five differential metabolites found in the A53T transgenic monkeys. The main relevant pathways for the two metabolites (Myristic acid and 3-Methylindole) were two-component system and bacterial chemotaxis, which were significantly higher in the control group (Fig. 4A, B). Metabolism pathways were the most significantly associated pathway in the three metabolites (Glyceric acid, p-Hydroxyphenylacetic acid, L-Aspartic) which was significantly increased in the A53T transgenic group (Fig. 4C, D). We found four common KOs from the metabolic pathway, including glyceraldehyde-3-phosphate dehydrogenase (KO0131), pyrimidine metabolism (KO0756), oxidative phosphorylation (KO2147), and benzoate degradation (KO4112) (Fig. 4F, I).

## Discussion

In our study, the composition and diversity of the gut microbiota are different in A53T monkeys of early PD and control monkeys. The results also confirmed previous reports that changes in gut microbiota are associated with changes in host genes^24^. In the present study, the alpha diversity significantly elevated in A53T transgenic monkeys compared to control monkeys (Shannon Index: *p* < 0.05). Similarly, the alpha diversity also significantly increased in PD patients compared to healthy people^25,26^. However, the alpha diversity significantly reduced in the rotenone-induced PD mouse model^27^. Therefore, the rodent model of PD induced by chemical drugs is obviously inconsistent with PD patients and A53T monkeys in the diversity of gut microbiota. The composition of gut microbiota and metabolites in A53T transgenic monkeys is very different compared to the normal monkeys by PCoA analysis.

In previous studies, Proteobacteria, Actinobacteria, Eggerthellaceae, and Sphingomonas have been observed to be significantly elevated in PD patients^26,28,29^. This trend is also observed in the A53T transgenic monkeys with early PD symptoms. Furthermore, Cupriavidus showed higher abundance in the non-tremor subtype of PD patients than in the tremor subtypes^25^, and Cupriavidus also showed similar high abundance in the A53T monkeys of early PD. Therefore, Cupriavidus may be a potential biomarker for early PD diagnosis. Prevotella was significantly decreased in PD patients^28,29^. Prevotella also significantly decreased in A53T monkeys. According to the previous study, Prevotella can decrease ratings for typical PD symptoms by produce hydrogen^30^. Across studies have also showed that Lactobacillus, Akkermansia, and Bifidobacteria enrichment are the most commonly identified taxa increased in human PD patients. In our study, Akkermansia was significantly increased in A53T monkeys compared to control monkeys (*p* = 0.03), which is consistent with the report showed that Akkermansia was significantly related to the greater presence of nonmotor symptoms^31^. Barichella et al. have found that Lactobacillus was significant positive associated with human PD patients with severe motor symptoms^32^, and Unger et al. found that Bifidobacteria were significantly more abundant in PD patients^33^. In our study, Bifidobacteria and Lactobacillus were increased in the A53T monkeys compared to control monkeys, but significant differences were not detected (*p* = 0.06 and 0.25, respectively), which might due to that the A53T monkeys were still young and showed mild early symptoms. We also observed that *Eggerthella lenta* significantly elevated in A53T transgenic monkeys, and *Eggerthella lenta* is a dopamine dehydroxylating strain that can convert dopamine to m-tyramine. M-tyramine is a chemical for regulates neurotransmitters, which was significantly elevated in the urine of patients with neurosis^34^. Odoribacter and Enterococcus were increased in PD patients^25^, but the abundance of two genera were lower in A53T transgenic monkeys compared to control monkeys. In Table 1, we summarized a comprehensive comparison of intestinal microbiota among Parkinson’s patients, A53T transgenic monkeys, and A53T transgenic mice, and in general, the monkeys at early PD showed consistent gut microbiota with human PD patients (Table 1).

**Table 1:** Comprehensive comparison of gut microbiota between Parkinson’s patients, A53T mice and A53T monkeys of early Parkinson’s disease.

	Parkinson’s patients	A53T monkeys	A53T mice
**Diversity**			
α-diversity	**↑**^26,32,48^	↑	/
β-diversity	clear separation^26,29,32,49,50^	clear separation	/
**Phylum**			
Proteobacteria	↑^33,49^	↑	↑^51^
Actinobacteria	↑^49^	↑	↑^51^
**Family**			
Eggerthellaceae	↑^26,29^	↑	/
**Genus**			
Akkermansia	↑^25,31,48,52–56^	↑	/
Prevotella	↓^30,32,52,53,56^	↓	↑^51,52^^,^
Bacteriodes	↑^25,28,29,55^	↓	↓^51^
Sphingomonas	↑^26^	↑	/
Odoribacter	↑^25,49^	↓	↓^51^
Cupriavidus	↑^52^	↑	/
**Species**			
Eggerthella lenta	↑^34^	↑	/

**Fig. 5:**
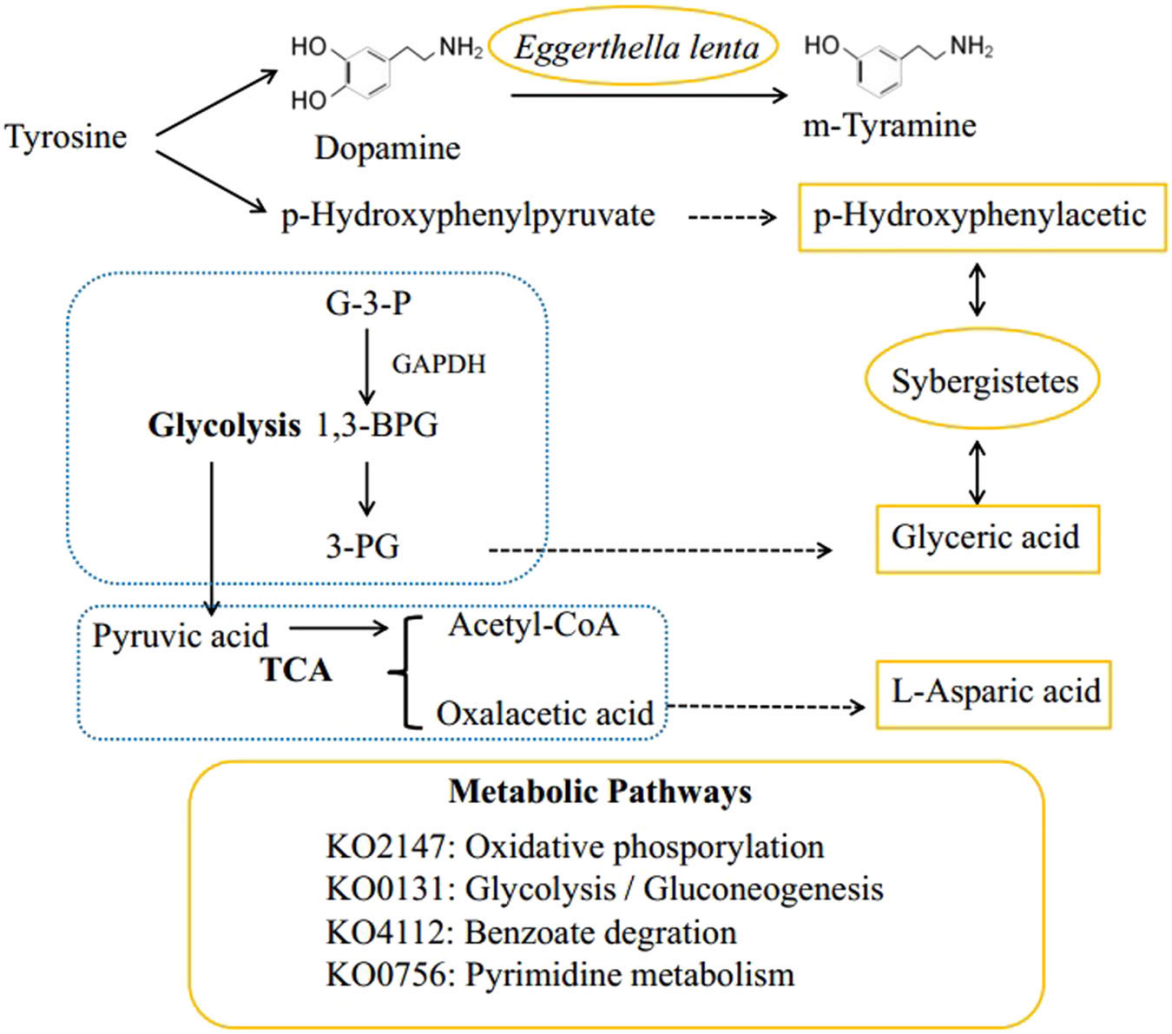
Metabolic pathway analysis. We speculate a possible metabolic response analysis of three differential metabolites, in which an increase of glyceric acid and L-aspartic acid may indicate an acceleration of glycolysis and TCA cycle, and functional analysis of differential metabolites also illustrates this speculated (KO2147, KO0131). In addition, tyrosine is decomposed into dopamine and p-hydroxyphenylpyruvate, *Eggerthella lenta* can convert dopamine to m-tyramine, p-hydroxyphenylpyruvate is oxidized to p-hydroxyphenylacetic. *Eggerthella lenta* and p-hydroxyphenylpyruvate was significantly increased in A53T monkeys. G-3-P: oxidation of plyeradehyde-3-phosphate, 1,3-BPG: 1,3-triglyceric acid diphosphate, 3-PG: 3-phosphoglycerate. Yellow: metabolites, microbiota, KO increased significantly in A53T monkeys.

Metabolites are the ultimate embodiment of cellular activity. Gene expression, splicing, and neuronal function in the brain can be regulated by small-molecule metabolites. A few previous studies have indicated that changes in short-chain fatty acids are associated with the onset of PD^33,35^. Our results also indicate that short-chain fatty acids (butyric acid, isovaleric acid, propionic acid, valeric acid, and isobutyric acid) in the A53T transgenic monkeys showed the trend of reduction compared to control monkeys. In the A53T monkeys, the glyceric acid in the colonic contents is significantly higher than that of control monkeys. A study has proven that the glyceric acid in the colonic contents of autistic mice also significantly elevated after transplantation of stool from autistic patients^36^. Glyceric acid is a product of glycolysis, which may indicate that the glycolysis process of A53T transgenic monkeys is accelerated, so a large amount of pyruvic acid may be produced. Pyruvic acid will be converted to acetyl-CoA lead to a corresponding amount of oxalacetic acid is required in the TCA cycle. A part of oxalacetic acid may be converted to L-aspartic acid. Accelerated glycolysis and the TCA cycle may indicate mitochondrial dysfunction in the A53T monkeys. Furthermore, tyrosine is decomposed into dopamine and p-hydroxyphenylpyruvate and p-Hydroxyphenylpyruvate is then oxidized to p-Hydroxyphenylacetic. p-Hydroxyphenylacetic acid also significantly elevated in A53T transgenic monkeys, which may cause loss of tyrosine in A53T monkeys. *Eggerthella lenta* was elevated in A53T transgenic monkeys, which can convert dopamine to m-tyramine. The speculated possible metabolic pathway and correlation are summarized in Fig. 5.

Analyze of metagenomics data allows us to explore possible pathways for three differential metabolites in A53T transgenic monkeys. Glyceraldehyde-3-phosphate dehydrogenase (GAPDH) (KO0131) is a key enzyme in glycolysis. The increase in concentration of this enzyme may accelerate the process of glycolysis and cause mitochondrial burden^37^. At the same time, GAPDH can trigger neuronal cell death by oxidative stress. Overexpression of GAPDH was also found in a mouse model of rotenone-induced PD, it can promotes neuron apoptosis in neurodegenerative disorders^38,39^. The oxidative phosphorylation reaction (KO2147) is increased in the A53T monkeys, which suggests that the mitochondrial energy supply accelerates and causes mitochondrial burden^40,41^. Previous study have shown that mitochondrial dysfunction caused by aberrant mitochondrial dynamics play an essential role in the pathogenesis of both sporadic and familial PD^42^. We speculate that a possible metabolic pathway is summarized in Fig. 5.

Furthermore, Myristic acid and 3-Methylindole were significantly decreased in the A53T monkeys compared with the control monkeys. Myristic acid is an enhancer of diacylglycerol kinase (DGK) δ expression in mouse. DGK is a lipid-metabolizing enzyme that phosphorylates diacylglycerol to produce phosphatidic acid. Decrease in Myristic acid production results in the deficiency of DGKδ, and subsequently induces obsessive-compulsive disorder (OCD)-like behavior through enhancing axon/neurite outgrowth in DGKδ-KO mice^43–45^. Myristic acid and 3-Methylindole were associated with ABC transporters, ABC transporters are the largest class of transporters widely found in bacteria and humans, which can hydrolyze ATP and provide energy to cells.

Previously, we generated the A53T transgenic monkeys that showed symptoms of early PD. As to our knowledge, there is no report studying the interaction between the PD and intestinal microbiota in nonhuman primates so far. Furthermore, whether the overexpress exogenous gene such A53T can drive the change of intestinal microbiota of monkeys is still unknown. Here, we reported the changes of intestinal microbiota and metabolites in the A53T monkeys, and the A53T monkeys showed very similar gut microbiota composition to human PD patients in a great extent. Therefore, our study suggested that the transgenic gene of A53T and α-syn aggregation may affect changes in microbiota and metabolites in rhesus monkeys, and the identified five compositional different metabolites that mainly associated with mitochondrial dysfunction may be related to the pathogenesis of PD. Our results will be beneficial for the mechanism study of α-syn aggregation induction of parkinsonism and the development of strategies of screen and diagnose of early stage of PD in human with taking the advantage of A53T transgenic nonhuman primate models.

## Methods

### Animals

A total of 10 rhesus monkeys were used in this study. Five transgenic A53T monkeys (5–7 years, three female and two male) generated in our previous study was assigned as A53T group^23^, and five normal monkeys (6–7 years, four female and one male) were used as normal control (Supplementary Table 3). All of the animals were individually caged. The animal room was set on a 12 h light:12 h darkness cycle. The temperature and the humidity of the animal room were kept at 18–26 °C and 40–70%, respectively. The animals were fed twice per day with commercial monkey chow (LabDiet, Harlan Laboratories, Inc., USA). Fresh fruits and vegetables (including apples, bananas, pears, onions, cabbage) were supplemented once per day. All procedures were approved by the Institutional Animal Care and Use Committee of Kunming University of Science and Technology, and were performed in accordance with the Guide for the Care and Use of Laboratory Animals (8th edition).

### Sample collection and DNA extraction

Fresh fecal samples were collected in sterile tubes from the 10 rhesus monkeys. Then, the fecal samples were transferred to the laboratory immediately in an ice bath and stored at −80 °C. The isolation of purified microbial genomic DNA was performed from each fecal sample using a MoBioPowerSoil® DNA Extraction Kit (Arlsbad, CA, USA) according to the manufacturer’s recommendation. The DNA concentration was measured using Qubit® DNA Assay Kit in Qubit® 2.0 Flurometer (Life Technologies, CA, USA).

### Library preparation for sequencing

Each sample needed a total amount of 700 ng DNA to be used as input material for the DNA sample preparations. According to the manufacturer’s recommendation, sequencing libraries were generated using NEB Next® Ultra DNA Library Prep Kit for Illumina® (NEB, USA), and index codes were added to attribute sequences for each sample.

### Clustering and sequencing

In the cBot Cluster Generation System, the clustering of the index-coded samples was performed by HiSeq 4000 PE Cluster Kit (Illumina) according to the manufacturer’s instructions. After cluster generation, the library preparations were sequenced on an Illumina Hiseq 4000 platform and 150 bp paired-end reads were generated.

### Analysis of fecal metabolites

The sample preparation and derivatization protocols were based on the method used in previously published procedures^46,47^. Samples were thawed on an ice bath to diminish sample degradation. Approximately 50 mg of each sample were homogenized with 300 μL of NaOH (1 M) solution using a homogenizer (BB24, Next Advance, Inc., Averill Park, NY, USA) and centrifuged at 4000 g and 4 °C for 20 min (Microfuge 20 R, Beckman Coulter, Inc., Indianapolis, IN, USA). After centrifugation, 200 μL supernatant from each sample was transferred into an autosampler vial (Agilent Technologies, Foster City, CA, USA), and the residue was further exacted with 200 μL of cold methanol. After the second step of homogenization and centrifugation, 167 μL supernatant from each sample was combined with the first supernatant from the same sample in the autosampler vial. The extracts in the autosampler vial were capped and submitted for automated sample derivatization with a robotic multipurpose sample MPS2 with dual heads (Gerstel, Muehlheim, Germany). Briefly, 20 µL of MCF was added to the mixture and the sample was vortexed vigorously for exactly 30 s. Another 20 µL of MCF was added for the derivatization a second time. Four hundred microliters of chloroform followed by 400 µL of sodium bicarbonate solution (50 mM) was added to achieve the separation. The prepared samples were centrifuged at 4 °C and 4000 g for 20 min, and the bottom chloroform layer was carefully transferred by the robotic preparation station to a capped empty autosampler vial preloaded with approximately 25 mg of anhydrous sodium sulfate. The sample pretreated with sodium sulfate was shaken on a laboratory shaker at 450 g and 4 °C for 20 min and further transferred to a capped empty autosampler vial for injection.

A gas chromatography coupled to a time-of-flight mass spectrometry (GC-TOFMS) system (Pegasus HT, Leco Corp., St. Joseph, MO, USA) operated in electron ionization (EI) mode was used to quantify the microbial metabolites in this project. Instrument optimization was performed every 24 h. The raw data generated by GC-TOFMS were processed using proprietary software XploreMET (v2.0, Metabo-Profile, Shanghai, China) for automatic baseline denoising, smoothing, peak picking, and peak signal alignment.

### Microbial metagenomic sequencing and metabolites bioinformatics analysis

A total of 10 fecal pellet samples from 10 monkeys were analyzed by metagenomics sequencing. We generated a total of 1,326,695,659 sequence reads with an average of 44,223,189 total reads per sample. The quality of sequencing data were examined using FASTQC (V 0.11.7) and MultiQC (V 1.7). 16 S ribosomal RNA from metagenomic data were filtered by SortMeRNA (V 2.1), and OTU was clustered in the Mothur (V 1.41.1) pipeline. Shotgun metagenomic reads were first trimmed and filtered to the host contamination using Trimmomatic (V 0.36) and Bowtie2as part of the KneadData (V0.6.1) pipeline (https://bitbucket.org/biobakery/kneaddata/wiki/Home). Kraken (V 1.0) and Bracken (V 1.0) was used to classify metagenomic sequences. Metagenomic sequences were assembled using Megahit (V 1.1.3) and QUAST (V 5.0.2) was used to check assembly quality. Coding sequences 40,461,161 were predicted by Prokka (V 1.12) from the metagenomic of the Megahit assembly. Among them, 1,817,945 sequences with amino acid lengths greater than 200 were merged by CD-HIT (V 4.7), and finally, the abundance calculation was performed using the Salmon (V 0.12.0) software to obtain 1,709,060 non-redundant coding sequences. The gene functional annotation was determined through ortholog assignment by eggNOG-mapper (V 1.0.3) (Supplementary Fig. 1).

### Integrated analysis of microbial metagenomic sequencing and metabolites

To show the relation between enriched KO and the abundances of metabolites, as well as microbial species, genus, and phylum, we calculated the Spearman coefficients between the abundances of metabolites and the counts of KOs, the abundances of microbial species, the abundances of the microbial genus, and the abundances of the microbial phylum. Furthermore, we also calculated the Spearman coefficients between the counts of KOs and the abundances of microbial species, the abundances of the microbial genus, and the abundances of the microbial phylum to show the enrichments of KOs in different microbial species, genus, and phylum.

### Statistical analysis

The experiments were not randomized. Statistical significance (*P* value < 0.05) was determined by using Two-tailed unpaired Student’s Test in MatLab (MathWorks, MA) with *t* test2 function, as appropriate and indicated in the figure legends and the main text. Spearman coefficients are used to measure correlations between the A53T and Control group with the corr function in MatLab. Figures only display representative results. PCoA results were visualized using ggplot2 package in R (3.5.1) for the first two principle components with 95% confidence interval. Errors and error bars represent SEM (standard error of mean) from five independent samples of each group.

### Reporting Summary

Further information on research design is available in the Nature Research Reporting Summary linked to this article.

## Supplementary information

Supplementary Information

Reporting Summary

## Data Availability

The obtained metagenomic profiles have been uploaded into the NCBI SRA database and are accessible via the accession number: PRJNA574851.
