# Supplementary material for: Gut microbiota and metabolites of α-synuclein transgenic monkey models with early stage of Parkinson’s disease
Source: NPJ Biofilms Microbiomes. 2021 Sep 2;7:69. doi: 10.1038/s41522-021-00242-3 (PMC8413421; doi:10.1038/s41522-021-00242-3)
Supplement: Supplementary file 1 — Supplementary Information [file 41522_2021_242_MOESM1_ESM.pdf]

Supplementary Table 1 Division of 14 strains that significantly increased in the A53T group

Species	Order	Phylum
Intestinimonas butyriciproducens	Clostridiales	Firmicutes
Flavonifractor plautii	Clostridiales	Firmicutes
Oscillibacter valericigenes	Clostridiales	Firmicutes
Christensenella massiliensis	Clostridiales	Firmicutes
Adlercreutzia equolifaciens	Eggerthellales	Actinobacteria
Slackia heliotrinireducens	Eggerthellales	Actinobacteria
Eggerthella lenta	Eggerthellales	Actinobacteria
Gordonibacter urolithinfaciens	Eggerthellales	Actinobacteria
Gordonibacte rmassiliensis	Eggerthellales	Actinobacteria
Eggerthella sp. YY7918	Eggerthellales	Actinobacteria
Magnetospirillum gryphiswaldense	Alphaproteobacteria	Proteobacteria
Rhodopseudomonas palustris	Alphaproteobacteria	Proteobacteria
Pannonibacter phragmitetus	Alphaproteobacteria	Proteobacteria
Rhodobacter sphaeroides	Alphaproteobacteria	Proteobacteria

Supplementary Table 2 We performed targeted metabolics analyses of colon contents from A53Ttransgenic and control monkeys, and a total of 93 metabolites were found.

Name	Control	A53T	p_value(t-test2)
Glyceric acid	302.852	2305.418	0.003084611
Myristic acid	89.35	23.778	0.024337375
L-Aspartic acid	271.78	693.658	0.028432144
p-Hydroxyphenylacetic acid	7.892	11.692	0.041063736
3-Methylindole	52.358	11.918	0.048309432
4-Hydroxybenzoic acid	7.248	11.858	0.06302988
4-Hydroxycinnamic acid	743.182	1487.61	0.065533686
L-Phenylalanine	142.554	60.242	0.06753077
Oxoglutaric acid	113.164	44.918	0.069050005
Hydroxypropionic acid	50.642	5.444	0.093155245
3-Indoleacetonitrile	6.758	13.574	0.093918939
1H-Indole-3-acetamide	10.222	0.526	0.106253005
Behenic acid	11.916	4.752	0.11138822
3-Hydroxybutyric acid	98.52	235.068	0.119707125
Glutathione	13294.064	3844.292	0.120989737
Adipic acid	4.498	7.51	0.121656987
Citric acid	151.788	267.814	0.123287569
5-Dodecenoic acid	33.762	19.3	0.125135998
Acetic acid	7120.696	4720.252	0.131692141
Pentadecanoic acid	206.902	123.882	0.133684791
Pelargonic acid	3.378	5.142	0.135627648
Isobutyric acid	99.296	162.562	0.138819812
3-Hydroxyphenylacetic acid	4.71	8.958	0.139132748
Malic acid	19.068	31.858	0.139168188
3-Hydroxyisovaleric acid	19.516	43.854	0.150815763
Hydroxyphenyllactic acid	205.514	365.46	0.161589369
Glycolic acid	13.152	9.52	0.164551518
L-Norleucine	44.45	25.588	0.193556532

L-Isoleucine	45.572	24.576	0.199325749
Methylsuccinic acid	2.542	4.324	0.200593407
Gamma-Aminobutyric acid	104.694	55.788	0.210707787
Tetracosanoic acid	10.882	5.474	0.223553971
Heptanoic acid	5.332	9.162	0.236186407
Nicotinic acid	4.296	3.106	0.244015835
L-Valine	63.822	38.568	0.244191334
L-Methionine	772.918	579.082	0.262899751
Malonic acid	5.6	12.714	0.269006315
L-Tyrosine	32.712	21.354	0.269685843
L-Leucine	69.896	46.398	0.271804894
Caproic acid	9.916	17.586	0.277747157
Glutaric acid	8.418	12.716	0.303666193
3-3-Hydroxyphenyl-3-hydroxypropanoic acid	1.402	3.34	0.308332935
Palmitoleic acid	1814.76	1345.794	0.31905468
Arachidonic acid	65.974	154.854	0.319711845
Propionic acid	730.138	189.654	0.319733965
Palmitic acid	629.66	468.442	0.322005236
8,11,14-Eicosatrienoic acid	38.566	48.666	0.32481524
Dodecanoic acid	34.374	27.188	0.325393437
2-Phenylpropionate	0.952	1.01	0.334079637
Capric acid	12.354	8.638	0.34615041
L-Homoserine	267.662	140.19	0.346304538
Arachidic acid	32.722	151.906	0.357361874
L-Alanine	2.516	2.102	0.35849681
Eicosenoic acid	35.974	106.874	0.37385979
Glycine	4.6	3.906	0.375257816
Stearic acid	390.98	254.208	0.375681283
Nonadecanoic acid	9.386	16.526	0.381839188
Oxoadipic acid	110.682	62.082	0.397328762
L-Glutamic acid	1340.046	1785.676	0.401546454

cis-Aconitic acid	12.362	13.996	0.45996535
L-Proline	56.746	89.696	0.469428016
Beta-Alanine	6.358	5.054	0.469538489
Phenylacetic acid	174.198	110.698	0.473145852
Oxalic acid	453.77	220.294	0.481638031
Docosapentaenoic acid n6	17.068	45.148	0.510002921
Vanillic acid	9.994	10.854	0.512494808
2-methyl-Butyric acid	147.828	132.14	0.520593311
Docosatrienoic acid	7.754	17.188	0.526755298
Isovaleric acid	145.486	128.956	0.531357669
3-Methyl-2-oxovaleric acid	248.614	265.574	0.600483883
Butyric acid	741.602	839.3	0.623792116
Erucic acid	8.15	9.936	0.666657791
Norvaline	8.72	12.85	0.675106048
Pyroglutamic acid	19.61	29.284	0.678023772
Fumaric acid	11.354	14.74	0.6981714
L-Lysine	133.324	160.336	0.698512415
Suberic acid	7.954	8.328	0.700445747
Linoleic acid	459.582	522.4	0.72509539
Succinic acid	12.294	14.768	0.727348136
L-Serine	69.522	60.122	0.749440992
L-Alpha-aminobutyric acid	1.36	1.544	0.794358045
Phenyllactic acid	1.376	1.268	0.829735249
Docosahexaenoic acid	16.368	19.708	0.842452142
Hydrocinnamic acid	49.65	52.716	0.844586326
Pimelic acid	6.374	6.91	0.850949343
Caprylic acid	4.378	4.024	0.861814725
Alpha-Linolenic acid	343.196	321.984	0.868830599
Valeric acid	206.5	211.042	0.886335696
Nervonic acid	132.112	144.974	0.889805407
D-2-Hydroxyglutaric acid	13.308	12.66	0.931870794

L-Asparagine	52.93	50.77	0.945259465
Heptadecanoic acid	53.216	51.898	0.948689465
Melatonin	23.732	23.788	0.972782961

Supplementary Table 3 General characteristics of the A53T transgenic and control monkeys.

	Animal	Group	Age (years)	Sex	Weight (Kg)
1	120714	Control	7	female	6.0
2	120718	Control	7	female	5.8
3	120720	Control	7	female	5.2
4	120708	Control	7	female	5.4
5	110807	Control	6	male	7.7
6	120666	A53T	6	female	5.3
7	130996	A53T	5.5	female	6.2
8	130916	A53T	5.5	female	5.7
9	110217	A53T	7	male	6.4
10	130919	A53T	5.5	male	5.0

Supplementary Figure 1

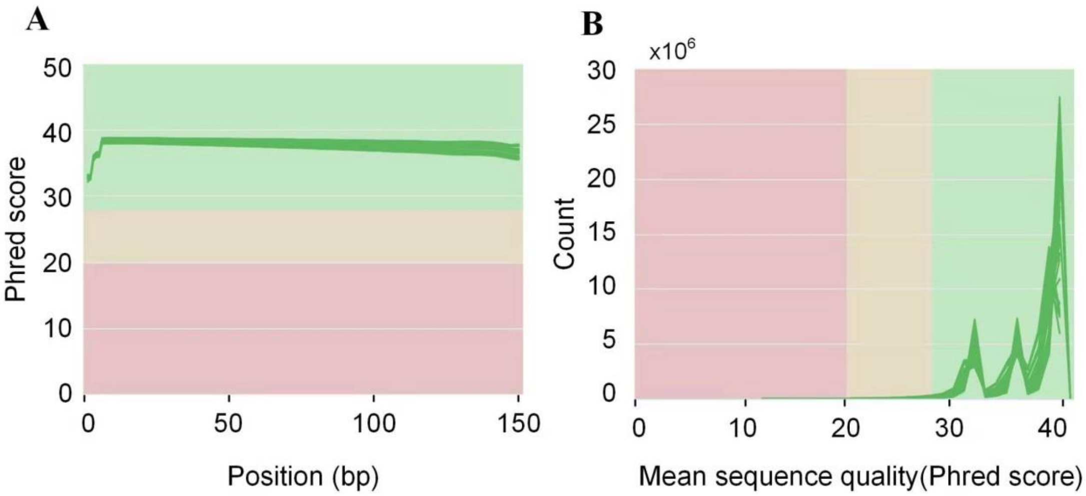

A. The mean quality value across each base position in the read, B. The number of reads with average quality scores
