# Supplementary material for: Gut microbiota and metabolites of α-synuclein transgenic monkey models with early stage of Parkinson’s disease
Source: NPJ Biofilms Microbiomes. 2021 Sep 2;7:69. doi: 10.1038/s41522-021-00242-3 (PMC8413421; doi:10.1038/s41522-021-00242-3)
Supplement: Supplementary file 2 — Reporting Summary [file 41522_2021_242_MOESM2_ESM.pdf]

nature portfolio

Corresponding author(s): Wei Si

Last updated by author(s): Aug 1, 2021

Reporting Summary

Nature Portfolio wishes to improve the reproducibility of the work that we publish. This form provides structure for consistency and transparency in reporting. For further information on Nature Portfolio policies, see our Editorial Policies and the Editorial Policy Checklist.

Statistics

For all statistical analyses, confirm that the following items are present in the figure legend, table legend, main text, or Methods section.

n/a	Confirmed	
☐	☒	The exact sample size (n) for each experimental group/condition, given as a discrete number and unit of measurement
☐	☒	A statement on whether measurements were taken from distinct samples or whether the same sample was measured repeatedly
☐	☒	The statistical test(s) used AND whether they are one- or two-sided<br>*Only common tests should be described solely by name; describe more complex techniques in the Methods section.*
☒	☐	A description of all covariates tested
☒	☐	A description of any assumptions or corrections, such as tests of normality and adjustment for multiple comparisons
☐	☒	A full description of the statistical parameters including central tendency (e.g. means) or other basic estimates (e.g. regression coefficient) AND variation (e.g. standard deviation) or associated estimates of uncertainty (e.g. confidence intervals)
☒	☐	For null hypothesis testing, the test statistic (e.g. F, t, r) with confidence intervals, effect sizes, degrees of freedom and P value noted<br>*Give P values as exact values whenever suitable.*
☒	☐	For Bayesian analysis, information on the choice of priors and Markov chain Monte Carlo settings
☒	☐	For hierarchical and complex designs, identification of the appropriate level for tests and full reporting of outcomes
☐	☒	Estimates of effect sizes (e.g. Cohen's d, Pearson's r), indicating how they were calculated

Our web collection on statistics for biologists contains articles on many of the points above.

Software and code

Policy information about availability of computer code

Data collection	No software was used
Data analysis	No software was used

For manuscripts utilizing custom algorithms or software that are central to the research but not yet described in published literature, software must be made available to editors and reviewers. We strongly encourage code deposition in a community repository (e.g. GitHub). See the Nature Portfolio guidelines for submitting code & software for further information.

Data

Policy information about availability of data

All manuscripts must include a data availability statement. This statement should provide the following information, where applicable:

- Accession codes, unique identifiers, or web links for publicly available datasets
- A description of any restrictions on data availability
- For clinical datasets or third party data, please ensure that the statement adheres to our policy

The obtained metagenomic profiles have been uploaded into the NCBI SRA database and are accessible via the accession number: PRJNA574851.

Field-specific reporting

Please select the one below that is the best fit for your research. If you are not sure, read the appropriate sections before making your selection.

☒ Life sciences ☐ Behavioural & social sciences ☐ Ecological, evolutionary & environmental sciences

For a reference copy of the document with all sections, see nature.com/documents/nr-reporting-summary-flat.pdf

Life sciences study design

All studies must disclose on these points even when the disclosure is negative.

Sample size	Research on non-human primates is inherently difficult, so only the 5 transgenic A53T monkeys (Niu et al.2015) generated in our previous study were assigned as A53T group. Correspondingly, we selected 5 normal monkeys that the age and weight are similar to A53T transgenic monkeys as the control group.
Data exclusions	In our study, no data were excluded from the analyses.
Replication	There are 5 monkeys in the A53T group and the control group. Because transgenic animals are very rare, it is difficult to repeat the experiment. But the investigator were blinded during data collection and analysis, so the reliability of data anlysis is guaranteed.
Randomization	5 transgenic A53T monkeys (Niu et al.2015) generated in our previous study were assigned as A53T group and 5 normal monkeys that the age and weight are similar to A53T transgenic monkeys as the control group.
Blinding	The investigator were blinded to group allocation during data collection and analysis.

Reporting for specific materials, systems and methods

We require information from authors about some types of materials, experimental systems and methods used in many studies. Here, indicate whether each material, system or method listed is relevant to your study. If you are not sure if a list item applies to your research, read the appropriate section before selecting a response.

Materials & experimental systems

n/a	Involved in the study	
☒	☐	Antibodies
☒	☐	Eukaryotic cell lines
☒	☐	Palaeontology and archaeology
☐	☒	Animals and other organisms
☒	☐	Human research participants
☒	☐	Clinical data
☒	☐	Dual use research of concern

Methods

n/a	Involved in the study	
☒	☐	ChIP-seq
☒	☐	Flow cytometry
☒	☐	MRI-based neuroimaging

Animals and other organisms

Policy information about studies involving animals; ARRIVE guidelines recommended for reporting animal research

Laboratory animals	A total of ten rhesus monkeys were used in this study. Five transgenic A53T monkeys (5-7years, 3 female and 2 male) and five normal monkeys (6-7years, 4 female and 1 male) were used as normal control
Wild animals	The study did not involve wild animals.
Field-collected samples	All of the animals were individually caged. The animal room was set on a 12 hours light: 12 hours darkness cycle. The temperature and the humidity of the animal room were kept at 18 °C to 26 °C and 40% to 70%, respectively. The animals were fed twice per day with commercial monkey chow (LabDiet, Harlan Laboratories, Inc., USA). Fresh fruits and vegetables (including apples, bananas, pears, onions, cabbage) were supplemented once per day. Stool was collected on the morning of the same date, and the samples were immediately stored in the -80°C refrigerator until testing.
Ethics oversight	All procedures were approved by the Institutional Animal Care and Use Committee of Kunming University of Science and Technology, and were performed in accordance with the Guide for the Care and Use of Laboratory Animals (8th edition).

Note that full information on the approval of the study protocol must also be provided in the manuscript.
